# Linking assessment to real life practice – comparing work based assessments and objective structured clinical examinations using mystery shopping

**DOI:** 10.1007/s10459-023-10284-1

**Published:** 2023-09-20

**Authors:** Angelina Lim, Sunanthiny Krishnan, Harjit Singh, Simon Furletti, Mahbub Sarkar, Derek Stewart, Daniel Malone

**Affiliations:** 1 Faculty of Pharmacy and Pharmaceutical Sciences, Monash University, 3052 Parkville, VIC Australia; 2 Department of Cardiovascular Sciences, University of Leicester, Glenfield Hospital, LE3 9QP Leicester, UK; 3 Monash Centre for Scholarship in Health Education, Faculty of Medicine and Nursing, Monash University, 3806 Clayton, VIC Australia; 4 College of Pharmacy, Qatar University, Doha, Qatar

**Keywords:** Objective structured clinical examinations, Work based assessments, Kane’s validity framework, Pharmacy

## Abstract

Objective Structured Clinical Examinations (OSCEs) and Work Based Assessments (WBAs) are the mainstays of assessing clinical competency in health professions’ education. Underpinned by the extrapolation inference in Kane’s Validity Framework, the purpose of this study is to determine whether OSCEs translate to real life performance by comparing students’ OSCE performance to their performance in real-life (as a WBA) using the same clinical scenario, and to understand factors that affect students’ performance. A sequential explanatory mixed methods approach where a grade comparison between students’ performance in their OSCE and WBA was performed. Students were third year pharmacy undergraduates on placement at a community pharmacy in 2022. The WBA was conducted by a simulated patient, unbeknownst to students and indistinguishable from a genuine patient, visiting the pharmacy asking for health advice. The simulated patient was referred to as a ‘mystery shopper’ and the process to ‘mystery shopping’ in this manuscript. Community pharmacy is an ideal setting for real-time observation and mystery shopping as staff can be accessed without appointment. The students’ provision of care and clinical knowledge was assessed by the mystery shopper using the same clinical checklist the student was assessed from in the OSCE. Students who had the WBA conducted were then invited to participate in semi-structured interviews to discuss their experiences in both settings. Overall, 92 mystery shopper (WBA) visits with students were conducted and 36 follow-up interviews were completed. The median WBA score was 41.7% [IQR 28.3] and significantly lower compared to the OSCE score 80.9% [IQR 19.0] in all participants (p < 0.001). Interviews revealed students knew they did not perform as well in the WBA compared to their OSCE, but reflected that they still need OSCEs to prepare them to manage real-life patients. Many students related their performance to how they perceived their role in OSCEs versus WBAs, and that OSCEs allowed them more autonomy to manage the patient as opposed to an unfamiliar workplace. As suggested by the activity theory, the performance of the student can be driven by their motivation which differed in the two contexts.

**Supplementary Information:**

The online version contains supplementary material available at 10.1007/s10459-023-10284-1.

## Introduction

Objective Structured Clinical Examination (OSCE) and Work Based Assessments (WBAs) are the two most valued assessments in health professional education to test the application of clinical knowledge in students (Harden & Gleeson, 1979; Miller, 1990; Norcini & Burch, 2007; Shumway & Harden, 2003). Despite the widespread use of OSCEs for the assessment of students’ clinical competence in health professions education for both formative and summative assessment purposes (Patrício et al., 2013), ongoing debate exists in the literature regarding the extent to which OSCEs authentically mirror real-life scenarios. OSCEs allow students to apply clinical knowledge to a simulated scenario, allowing them to make consequence free mistakes compared to a real patient. OSCEs allow educators to assess a range of skills within one scenario such as communication, interpersonal skills and problem solving (Khan et al., 2013). OSCEs are also a robust tool of assessment which involve case development processes, standardized and objective evaluation, examiner and simulated patient training, and are reproducible. Although a robust assessment tool, some argue OSCEs carry a risk of making the encounter artificial and far removed from actual clinical practice (Brydges et al., 2015; Gormley et al., 2012; Weersink et al., 2019). Implicitly, the lack of veracity in resembling real practice may encourage students to merely memorize and go through the ‘motions’ of the OSCE checklist rather than learning and demonstrating deeper understanding of the intended skills particularly on the more humanistic aspects of the interaction (Boursicot et al., 2011; Gormley, 2011). Others have raised concerns that OSCEs are designed such that they only assess observable behavior, rather than a student’s cognitive skills such as their reasoning process, and therefore they may not be reliable assessments (Witheridge et al., 2019). Another point of concern is the reliability of OSCEs as an objective assessment as this relies on assessors marking equally, to minimize variation and lessen the potential impact on student outcomes (Malau-Aduli et al., 2022) which does not allow the flexibility for unpredictable real-life situations. Furthermore, it is important to consider the practicality of the assessment, including the logistics of exam preparation and the accommodation of large groups of students (Malau-Aduli et al., 2022).

Assessments should be purposeful, fair and robust and extrapolate to preparing students for practice. Kane’s validity framework (Kane, 1992) provides a comprehensive approach for evaluating the validity evidence of assessment methods (Hess & Kvern, 2021), encompassing aspects such as scoring, generalization, extrapolation and implications of assessments. Scoring should be purposeful and accurate, with clear evidence supporting the generalizability of scores. OSCE scores should accurately infer the expected competencies of real-life practice, and judgements made about the ability of learners should be made correctly to ensure their readiness for practice. When applied to OSCEs in pharmacy education, Kane’s framework encourages educators to ensure that OSCEs measure relevant content, are conducted in a standardized manner, have clear and consistent scoring rubrics, and demonstrate meaningful consequences in terms of predicting real-world pharmacy practice (Kane, 1992). Whilst the scoring and generalizability have been discussed in the literature; limited studies on the extrapolation inference in Kane’s validity framework and its implications for assessing the overall validity of the OSCEs has been explored.

The debate on the authenticity of the OSCEs has highlighted the need to explore alternative approaches, such as WBAs. WBAs are part of work-integrated learning which is an approach to enhance students’ learning experience, providing opportunities for them to practice and apply their knowledge and skills in real-world situations (Bartlett et al., 2023). WBAs are purposeful and serve a critical role in clinical training as the assessments are authentic in terms of involving day-to-day patient care (Norcini & Burch, 2007). WBAs have also shown to serve an important role in providing feedback to learners and supporting further learning (Henry & West, 2019; van der Vleuten et al., 2012) but also have its disadvantages of inconsistency of feedback, variation in workplace supervisor training and environment challenges. WBAs and OSCEs are both widely used for the assessment of clinical competence in health education, however, there has not been any literature comparing the two contexts and how students perform in a simulated versus un-simulated environment. Real life patients are commonly used in WBAs to assess competencies in the workplace (Khalife et al., 2022), and have found to be useful to have an authentic voice and provide more valuable feedback that prepare students for practice (Malau-Aduli, 2022). Our WBA used simulated patients as mystery shoppers as our study aims to compare the same clinical case in OSCEs and WBAs. Therefore, this study aims to compare the performance of students in an OSCE to their performance in real-life (as a WBA) using the same clinical scenario, and to understand what factors affect a student’s performance in a real life setting to inform future assessments.

## Methods

### Context

Underpinned by the extrapolation inference in Kane’s validity Framework, the purpose of this study is to link assessment to real life performance. At our institute, third year pharmacy students complete an infectious diseases OSCE at the end of Semester 1 (in May). In this OSCE, there were three stations (Hospital, Counseling and Primary Care) which were each eight minutes in duration. Students needed to complete and pass all three stations to pass the OSCE. Further details on the specifics of the OSCE can be found in Assessment of Antimicrobial Stewardship through objective structured clinical examination in pharmacy education (Lim et al., 2023). The hospital station required reviewing a hospital drug chart in the hospital setting, the counseling station required counseling on a new prescription in the community pharmacy setting, and the primary care station involved answering a consumer query or product request in the community pharmacy setting. Examinable topics across all OSCE stations included Skin and Soft Tissue Infections, Community Acquired Pneumonia, Antibiotic allergy, Human Immunodeficiency Virus, Hepatitis B and C, Ear infections, Urinary Tract Infections and Sexually Transmitted Diseases. These topics were spread across the sessions so different students encountered different topics. Students were marked by analytical clinical checklists as well as communication rubrics. Cases have been piloted and validated by practicing pharmacists and standard setting was performed for the rubrics. In addition to this, third year pharmacy students complete a 4 week student placement in a community pharmacy in Semester 2 (in September or November), where they are supervised by a workplace supervisor who is a registered pharmacist. During this placement, a student performs tasks a pharmacist can carry out in day to day activities but under supervision.

### Study design

This research was guided by pragmatism, which acknowledges the importance of both objective and subjective evidence in understanding reality (Johnson & Onwuegbuzie, 2004). Aligning with this view, a sequential explanatory mixed methods approach was used to not only determine how students perform in both assessments, but also to understand the reasons behind their performance as learners can have bespoke experiences. Quantitative data were obtained by assessing students’ real-life performance through a method referred to as ‘mystery shopping.‘ This method involves the use of a simulated patient, referred to as a ‘mystery shopper,‘ who is intricately trained to seamlessly emulate authentic patient interactions. This ‘mystery shopping’ method, widely employed in health services research, is particularly effective in natural work settings (Collins et al., 2021). Mystery shopping took place within a community pharmacy that provided a platform for genuine observation without the constraints of prior appointment, which is often the case with other healthcare professionals (Alexander, 1991). Mystery shopping used simulated patients who are trained to enact predetermined scenarios, and pretend to be patients asking for advice and should be indistinguishable from genuine patients, to assess aspects of customer service or provision of care (Mesquita et al., 2010; Watson et al., 2009). The simulated patients who visited students on their WBA were not known to the students and would not have been recognizable by students as any different from a genuine customer. Mystery shopping is an unobtrusive means of observing actual staff responses in a natural environment, under conditions uninfluenced by awareness that behavior is being monitored (Puspitasari et al., 2009), thus eliminating the Hawthorne effect. It is thus an effective method of deriving valid, true-to-life outcomes, which are otherwise challenging to achieve by any other method (Watson et al., 2006). Mystery shoppers either asked to purchase an over the counter product or had a customer query that would lend itself to either a referral or be recommended a product. Mystery shoppers in this study did not purchase any products but instead would tell the student they would come back later to purchase the product.

It was only possible to conduct this study around one station (the primary care station) as the hospital station was set in a hospital setting and the counseling station requires a real patient prescription. Unlike hospital wards, community pharmacies are accessible to the general public and mystery shoppers can easily enter the premises and are able to replicate an OSCE case in real life and be indistinguishable from a real patient.

**Fig. 1:**
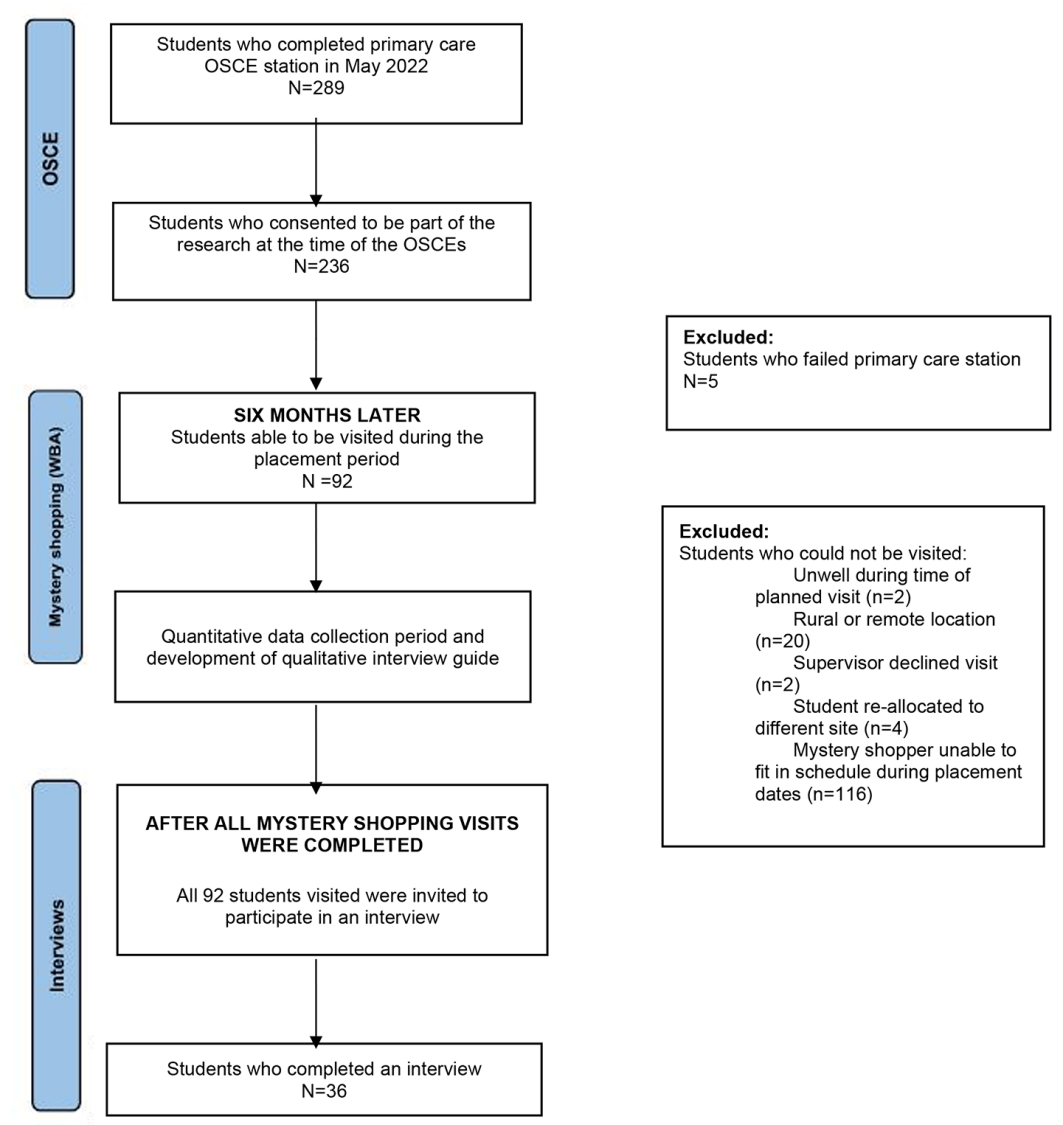
Study Timeline

Mystery shoppers visited pharmacy students at their student placement worksite in 2022 (Semester 2), about 4–6 months after their OSCE (Semester 1, 2022), simulated the same case scenario they were given in their OSCE, and marked students with the same rubrics. Figure 1 shows the timeline of the study and total number of participants at each stage. A total of four mystery shoppers were used to reach as many students as possible during the student placement period. Mystery shoppers are trained simulated patients who have been used in OSCEs previously at the faculty but were not used in the OSCE related to these participants so were unfamiliar to the participants. After all the WBAs (Mystery shopping visits) for this study were completed, it was revealed to students that they had a mystery shopper visit from the faculty in the form of a WBA, and students were invited to participate in a semi-structured interview to reflect on their experience and discuss their performance. This study was conducted according to the guidelines of the Declaration of Helsinki and approved by the Monash University Human Ethics Low Risk Review Committee (Project ID 32,537).

### Participants and setting

This study was conducted at a tertiary institution in Melbourne, Australia. All third year pharmacy undergraduate students who participated in the OSCE (298 students) were invited to participate at the beginning of the year. Of the 298, 236 consent to participate (Fig. 1). As part of the informed consent process, students were informed that a mystery shopper would visit them on their student placement and assess them, but they would not know when and who. After the consent process, mystery shoppers randomly selected participants to visit based on suitable location and time in discussion with their workplace supervisor. Students who failed the OSCE were excluded from participating as these students are given remediation tasks and extra support before their OSCE resit, thus would not be representative of a typical student in the cohort.

### Quantitative data collection

Prior to each mystery shopper visit, mystery shoppers (trained simulated patients) liaised with workplace supervisors regarding the visit and requested workplace supervisors not interfere when the student is being assessed. Workplace supervisors were asked to ensure students were accessible to the mystery shopper during the time of the visit and to not disclose when the visit was occurring. Mystery shoppers were given the same case guide to play the same case as the actual OSCE and given the same rubrics to mark their performance. Visits were audio recorded. In addition, student demographics such as gender, graduate entry, international status as well as experience in part time work were also collected through enrolment data. Some of these factors have been shown to be predictors of student success in clinical examinations in pharmacy (Lyons et al., 2020). Graduate entry students are students who have completed a previous science degree that can be offered a direct entry into the pharmacy undergraduate degree in its third year provided they complete the necessary bridging courses (Caliph et al., 2022).

### Qualitative data collection

After all the visits were conducted, the visits were revealed to the students and students were invited to participate in a semi-structured interview discussing their performance and comparing their experience with this mystery shopper encounter in a work based environment to their OSCE in a simulated environment. Interviews were approximately twenty minutes long and conducted over Zoom by the one research assistant. Each interview was recorded and transcribed, and ranged from 15 to 20 min in length. Transcripts were then cross-checked with the audio recording for accuracy. Interviews were guided by a semi-structured interview guide (Appendix 1). The interview guide consisted of questions asking them to compare their experience and performance in the mystery shopper encounter compared to their OSCE encounter and to describe factors that influenced their performance in each context. Students were also given feedback about their performance in the mystery shopper encounter. Prior to the interview, the interviewer listened to the recording of the mystery shopping visit (WBA) of the student and made notes in regards to any poor performance factors they wanted to explore in the interview.

### Data integration and analysis

Quantitative data were analyzed using SPSS version 26 (SPSS Inc., Chicago, IL). Data were not normally distributed and hence non-parametric tests were used. A Wilcoxon Signed Rank Test was used to compare students’ mystery shopping scores with their OSCE scores. A Kruskal-Wallis Test was used to compare the scores between different categories within the OSCE and mystery shopping group. A p-value less than 0.05 was considered statistically significant. Correlations between OSCE and WBA scores were measured using Pearson’s correlation coefficient.

In line with sequential explanatory design, after quantitative data were analyzed, qualitative interview guides were further developed to explain the discrepancy between OSCEs and WBAs grades, linking to variations in the scores on their marking rubrics. This mainly included exploring the different components of the marking rubric history taking and disease management. Since there was a huge discrepancy in the marks between OSCEs and WBAs; it was decided by the research team to explore knowledge retention before exploring factors that influenced performance. Conversations with the mystery shoppers occurred in tandem to ensure we account for their perceptions on influencers of performance. An example of this was mystery shoppers noting students shied away from discussing certain topics and they suspected they didn’t really have a knowledge gap clinically but perhaps were embarrassed by the presenting complaint. A final interview guide has been provided in Appendix 1. Prompts in question 5 of the interview guide were mainly guided by the feedback from the mystery shoppers as well as the rubrics.

For qualitative data, NVivo (QSR International, Version 1.3) was used to manage the coding. A thematic analysis approach was used to analyze the data, which involved identifying patterns and themes that developed from the rounds of analysis.(Maguire & Delahunt, 2017) The analysis was carried out by four investigators who initially read the first four interview transcripts independently then met to discuss and generate a list of codes.(Braun & Clarke, 2006) In the second round, two researchers coded two interviews independently using the generated list of codes, and then compared and discussed their findings to arrive at a consensus for a final codebook. The individual coding files were merged to create a final coding scheme, and the codes were categorized to generate key themes. The researchers continued coding interviews until they reached satisfactory information power. To ensure intercoder reliability, researchers calculated Cohen’s kappa after the first two rounds. Cohen’s kappa for intercoder reliability was 0.82, signifying almost perfect agreement between coders (Viera & Garrett, 2005). Finally, the remaining interviews were coded by one researcher using the final codebook.

### Team reflexivity

Authors (AL, HS, DM and SF) conducted the majority of the thematic analysis. These authors have previous experience in thematic analysis, are all academics who teach clinical content at the faculty and also practicing pharmacists. At the first phase of coding, the team conducted a team reflexivity exercise by discussing what they believe a good assessment should entail and what their thoughts were on OSCEs and WBAs in regards to preparation for practice. In this discussion, we believe all authors had positive beliefs to both OSCEs and WBAs and there were no inherent biases to a particular assessment.

## Results

### Quantitative data

**Fig. 2:**
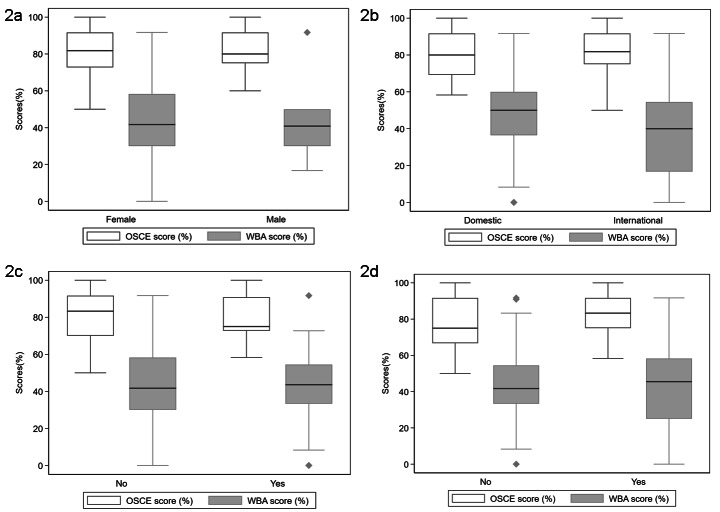
Comparison of student Characteristics across OSCE and Mystery shopping (WBA) performance: **2a**) Gender; **2b**) Student category (Domestic/International Status); **2c**) Graduate entry status; **2d**) Previous or current work experience

A total of 92 students were visited by our mystery shoppers. The original cohort size was 289; 5 students were ineligible as they failed the primary care station in the OSCE. Overall, students’ WBA (mystery shopping) scores were significantly lower compared to their original OSCE scores. The median mystery shopping (WBA) score was 41.7% and significantly lower compared to the OSCE score (80.9%) in all participants (p < 0.001). Students performed poorly in their mystery shopping encounters across both genders, irrespective of their prior or current experience working in pharmacies, whether they were a graduate entry or international student. Although domestic students recorded a slightly higher score compared to their international counterparts, it was still significantly lower compared to their actual OSCE scores (Table 1). For OSCE scores, the standard Deviation (SD) was 12.5%, with a range of 50–100%. For the WBA score, the standard deviation was higher (22.6%), with a greater range (0-91.7%). Figure 2 depicts the spread between these main four characteristics.

**Table 1:** OSCE scores *versus* mystery shopping (WBA) scores by student characteristics

Characteristic	N (%)	Median Score (IQR), %		*p*-value^a^
		OSCE Score	Mystery Shopping (WBA) Score	
**All participants**	92 (100)	80.9 (19.0)	41.7 (28.3)	< 0.001
**Gender**				
Male	10 (10.9)	80.0 (20.0)	40.9 (23.0)	0.005
Female	82 (89.1)	81.8 (19.7)	41.7 (30.0)	< 0.001
**Experience Working in Pharmacy**				
Yes	53 (57.6)	83.3 (17.9)	45.5 (34.2)	< 0.001
No	39 (42.4)	75.0 (25.0)	41.7 (21.2)	< 0.001
**Graduate Entry**				
Yes	26 (28.3)	75.0 (18.2)	43.6 (25.4)	< 0.001
No	66 (71.7)	83.3 (21.7)	41.7 (30.0)	< 0.001
**Student Category**				
Domestic	47 (51.1)	80.0 (22.5)	50.0 (23.6)	< 0.001
International	45 (48.9)	81.8 (17.9)	40.0 (37.8)	< 0.001

^a^ Comparison of scores between OSCE and Mystery shopping

**Fig. 3:**
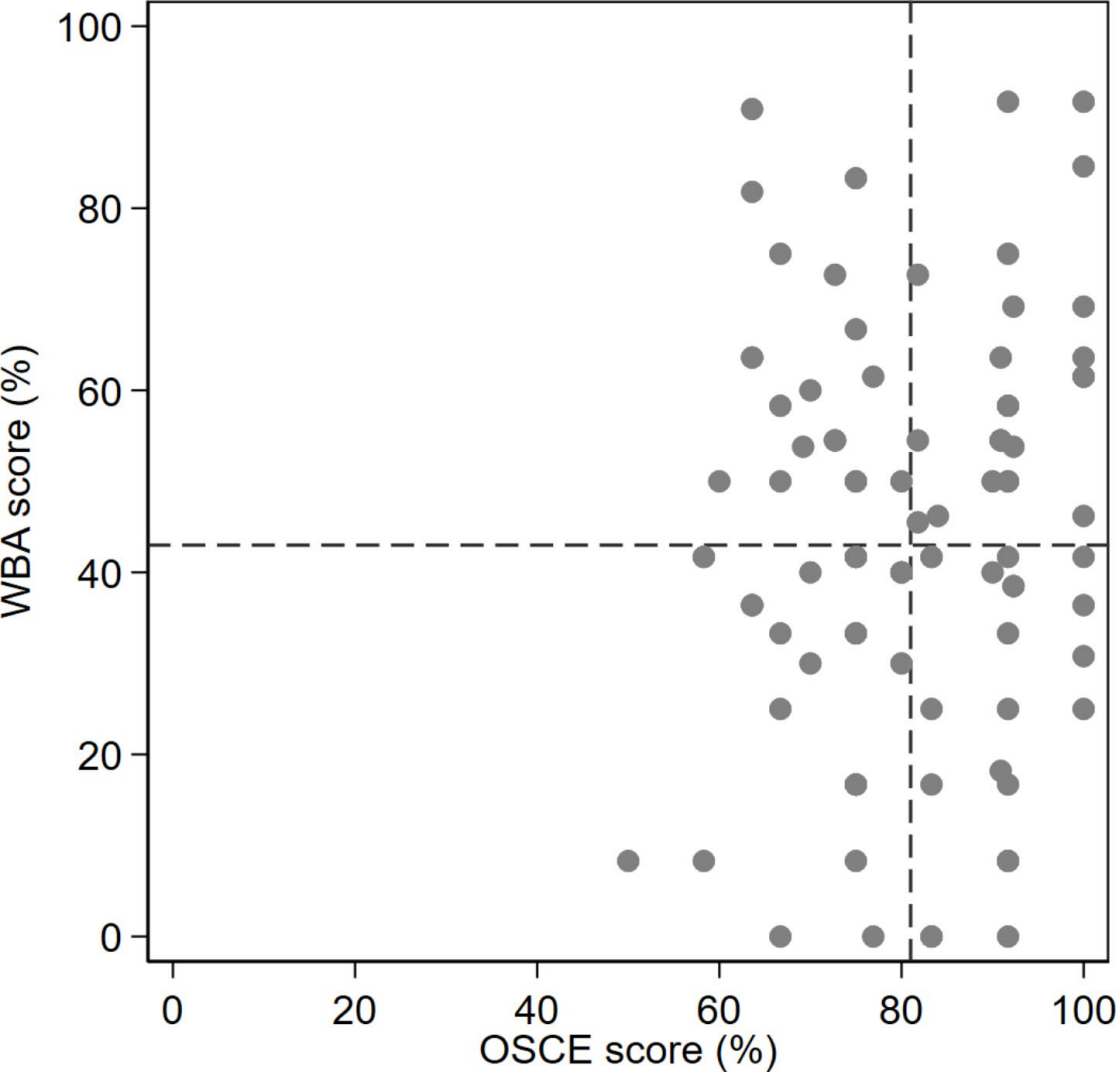
Comparison of OSCE performance versus mystery shopper (WBA) performance (N = 92)

A subgroup analysis was performed based on students’ grades in their actual OSCE where students were categorized into tertiles according of high performing (category 1: 90–100%), moderate performing (category 2: 70–89%) and low performing (category 3: 50–69%) (Table 2). Students’ performance was significantly lower in the mystery shopping (WBA) encounter regardless of whether they were a high performing or low performing student in the OSCE. Although high-achievers (i.e. those who scored ≥ 90% in the OSCE) had recorded a slightly better score in the mystery shopping visits compared to students from other categories, this difference was not statistically significant (p = 0.327) (Table 2). In addition, after conducting a linear correlation test between OSCE and WBA score, no correlation was found, r(91) = 0.09, p = 0.38. Figure 3 showcases the spread of scores in the OSCE compared to the WBA. When the results of the cohort were divided into tertiles of categories of performance (as per categories in Table 2) according to their OSCE score, the higher tertile group (median = 92.3, range 91.7–100) tends to have a positive correlation with their WBA score (r(28) = 0.31, p = 0.1), suggesting that performance of high OSCE scoring students may have some translation to performance in real life. No correlations presented in the other two tertiles.

**Table 2:** Subgroup comparison of students’ performance in mystery shopping (WBA) visits based on their OSCE scores

Categories based on OSCE Scores	N	OSCE Score (%)		Mystery Shopping (WBA) Score (%)		*p*-value^d^
		Median (IQR)	*p*-value^b^	Median (IQR)	*p*-value^c^	
Category 1 (90–100%)	36	91.7 (8.3)	< 0.05	50.0 (30.1)	0.327	< 0.001
Category 2 (70–89%)	37	75.0 (6.8)		41.7 (31.4)		< 0.001
Category 3 (50–69%)	19	63.6 (6.7)		41.7 (30.3)		0.009

^b^ Comparison of scores within categories (OSCE).

^c^ Comparison of scores within categories (Mystery shopping WBA).

^d^ Comparison of scores between OSCE and Mystery shopping (WBA).

When comparing students’ performance in specific components of the marking rubric, mystery shopping scores were significantly lower in all aspects. Of note, students’ performance in information gathering during the mystery shopping encounters was particularly low (i.e., 40%), scoring very little questioning in real patients. (Table 3)

**Table 3:** Comparison between OSCE scores and mystery shopping (WBA) based on specific components of the marking rubric

Components of Marking Rubric	Median Score (IQR), %		*p*-value^e^
	OSCE Score (N = 92)	Mystery Shopping (WBA) Score (N = 92)	
Information Gathering	85.7 (25.0)	40.0 (32.1)	< 0.001
Disease Management	66.7 (33.3)	55.0 (41.7)	< 0.001
Communication Skills	81.0 (33.3)	71.4 (27.4)	< 0.001

^e^ Comparison of scores between OSCE and Mystery shopping (WBA)

**Fig. 4:**
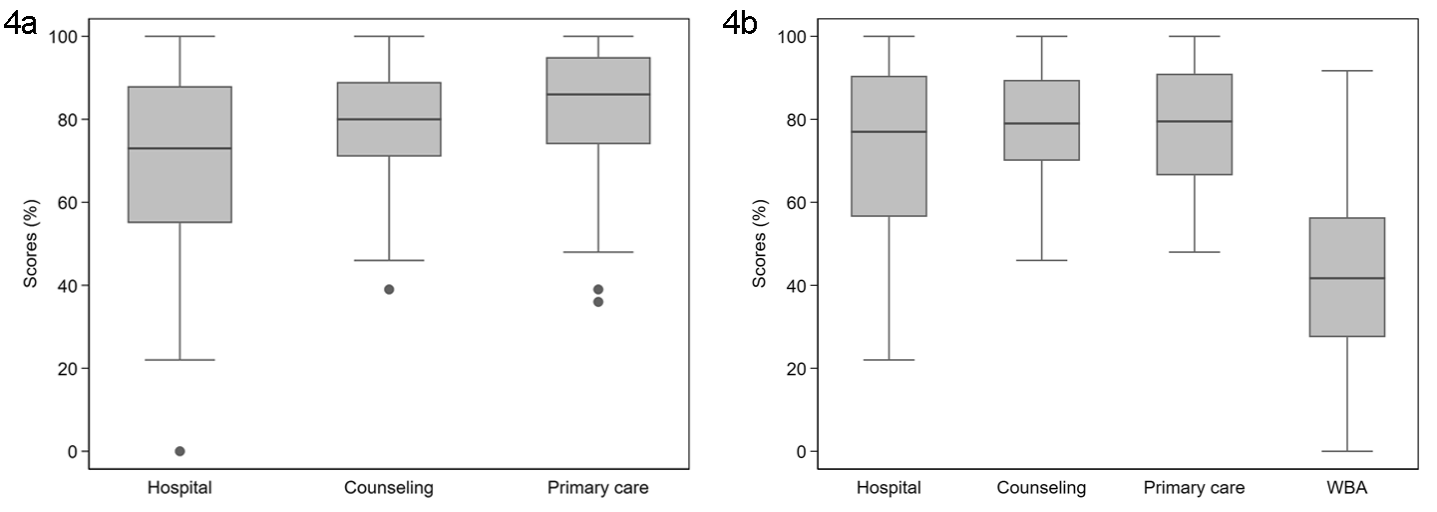
Comparison of the whole learner cohort across all three OSCE stations (N = 289): 4a) OSCE scores for all three stations (N = 289); 4b) OSCE scores for included cohort who had mystery shopping visits (N = 92)

When comparing the cohort of 92 to their peers in their original OSCE assessment and other stations; this cohort was not distinct in any way and performed similarly to their peers (Fig. 4). In the original cohort of 289 students, compared to other stations, the ‘primary care’ station was easier, with a median score of 86 [IQR: 74–95]. This is higher than the ‘hospital’ stations scored at median 73 [IQR: 55–88], and the ‘counseling’ stations scored at median 80 [IQR: 71–89], respectively. In the cohort of 92 students included in the manuscript, their performance in the ‘primary care’ station was comparable to their performance in the ‘hospital’ and ‘counseling’ station (Fig. 4).

### Qualitative data

Thirty-six students out of the ninety two consented to interviews; all 36 participants who consented to interviews were interviewed. Through thematic analysis of the interview transcripts across all 36 transcripts, three primary themes were generated: ‘design’, ‘environment’ and ‘characteristics’ (Table 4). Subcategories within each theme are italicized in the following text, and a quotation is provided for each subcategory. The interviews were numbered, and the number is given after each quotation. Exemplar quotes for each subcategory are included in Appendix 2.

**Table 4:** Themes and Subthemes

Main Theme	Subtheme
The fundamental design of the OSCEs differ from real life	Assessment preparation is different from preparing for practice
	Case realism was achieved and the assessment cases do reflect real life cases
	The exam environment is not realistic
	Feedback from a real-life scenario is perceived as more beneficial than feedback from an assessment
	The simulated patient is still perceived to be not a real patient
The work environment has intrinsic factors impacting on the student behavior and performance, meaning simulation can never be fully replicated	The work supervisor or team can impact on WBA performance
	Their perceived role in the workplace can impact on WBA performance
Students have individual learning characteristics that affect the change in performance in WBA and OSCEs	Students who are concerned about their communication skills are even more impacted in real life
	Students who have had previous or current work experience in pharmacy feel more confident with dealing with real life cases
	Students are still unfamiliar with teaching content even after the assessment

### The fundamental design of the OSCEs differ from real life

Students found that preparing for WBAs was different from preparing for the OSCE highlighting that *assessment preparation was different from preparing for practice*. While some felt the need to memorize information for the OSCE, they found that such memorization was not as crucial for practice. Structured interaction with simulated patients during the OSCE was noted to be more stressful than real-life encounters, but real-life interactions also raised concerns about forgetting important questions. Students suggested that OSCEs and WBAs should be combined to ensure comprehensive preparation for practice.

*“I found in the OSCE I’m more prompted to ask, like the referral questions. But in real life I forgot about asking the referral questions, because when I asked about the symptoms they already sounded like referral symptoms, so I didn’t like bother checking if they had other referral symptoms” [35]*.

Students highlighted how closely the OSCE cases mirrored real-life scenarios and found *case realism was achieved and the assessment cases do reflect real-life cases, stating that OSCEs were* beneficial in preparing for actual patient interactions. They believed the knowledge and skills gained from the OSCE could be applied to help individuals in real-life situations.

*“I feel like it[WBA] was the same case as my OSCE…wthich I can remember off the top of my head” [30]*.

While many students had positive feedback, some highlighted that *the OSCE exam environment is not realistic*. They felt that the pressure associated with the OSCE due to the exam conditions is not the same as real-life interactions with patients, which have fewer time pressures and more freedom in directing patients towards products.

*“I think the only thing that made me more confident was the fact that I was sort of just not under exam pressure… I could sort of take control of how I wanted to run that interaction….I could do it the way that I wanted to do it rather than you know what sort of was expected of you in an exam situation. So I think that was the only thing that made it easier, and made my performance better…”[13]*.

Students acknowledged that *feedback from real-life situations is more valuable* than assessment feedback. However, they understood the difficulty in providing real-life feedback consistently. Nevertheless, they valued real-life feedback for its patient perspective on their performance.

*“I think it’s helpful. I guess I can see what my performance is during placement…I asked the pharmacist for feedback. But like the patient’s perspective… that’s kind of important. Yeah” [15]*.

Lastly, students expressed that *the simulated patient is still perceived to be not a real patient*. This indicated that students felt that the OSCE simulated patient did not feel real, whereas in real-life scenarios, there was a higher level of responsibility when making therapeutic decisions that could affect patients. In these cases, it was crucial to provide accurate information to patients, as any mistakes could have serious consequences.

*“ In a real life situation, I think it was just a lot more natural… I’m not going to get assessed on this, but I need to make sure that this patient is still getting the correct information, because that is a lot more important than getting a grade… you want to be providing the right information to patients…so I suppose in that aspect. It [WBA] was a little bit more serious” [10]*.

### The work environment has intrinsic factors impacting on the student behavior and performance, meaning simulation can never be fully replicated

During the interviews, students expressed the approachability and positivity of the work *supervisor or team can impact on WBA performance*. Students mentioned in a WBA, they could seek advice from if they were unsure about their clinical decisions which helped them feel more confident when providing counseling.

*“I still feel a little bit unsure of things, just double checking. I feel like I know already, but I just sometimes need to double check with the pharmacists, and helped confirm my decision, or else i’d be much more hesitant…”[17]*.

Students reported that *their perceived role in the workplace can impact on WBA performance*, leading to feelings of incompetence during WBAs. Patients may trust the pharmacist more, and students may feel temporary and unimportant in the workplace.

*“There were customers that were aggressive because they know I’m still a student…they didn’t really respect the students. Then they insisted on speaking to the pharmacists, because they know I’m just a student, and they don’t talk to me…”[33]*.

### Students have individual learning characteristics that affect the change in performance in WBA and OSCEs

Interviews revealed that students who were *concerned about their communication skills are even more impacted in real life*, specifically when interacting with patients.

*“I’m still not really that confident in counseling in English and am worried patients won’t understand me and will do the wrong thing” [36]*.

On the other hand, *students who have had previous or current work experience in pharmacy feel more confident with dealing with real life cases*. Some students noted the benefits of work experience in pharmacy for WBAs, as they have already had many opportunities to practice interacting with patients at their usual workplace.

*“I think when I’m working like in my regular community pharmacy job. I definitely have more opportunities to speak to patients. But on placement I didn’t have as many opportunities. Yeah, I guess it’s hard because you sort of there for a short period of time, and depending on the time of year. If they’re super busy, you know it’s a little bit harder to [get support]. But i’m glad I had some”[22]*.

*Students are still unfamiliar with teaching content even after the assessment with* some students expressing uncertainty about the content even after attending lectures, receiving preparatory materials, and completing an OSCE. Instead of attending to patient queries independently, they sought to confirm their recommendations with resources or pharmacists during WBAs to ensure patient safety.

*“I still feel a little bit unsure of things…I just like to double check with the pharmacists” [17]*.

*“I had to like research my stuff, I was like, oh, I’ll double check because. I was probably 90% confident, but it was like I should double check, because she’s pregnant and I need my recommendations to be safe” [24]*.

## Discussion

The primary finding from this study was that pharmacy student performance in OSCEs did not extrapolate to real life practice (assessed as a mystery shopping WBA). A significant difference was shown in information gathering between OSCEs and WBAs. Student characteristics that have been shown to be predictors of success in clinical examinations such as graduate entry, domestic students, previous or current work experience were not sure to affect performance between OSCEs and WBAs in our study (Caliph et al., 2022; Lyons et al., 2020). Despite this, students highly regard both assessments, reflecting on the fact they complement each other and are both needed to consolidate their learning. Students commented in the interviews that the preparation for the OSCE consists of memorizing an abundance of information that would not be necessarily applicable to all real-life scenarios. This aligns with other studies which have found OSCEs focus on memorization of specific tasks and procedures rather than on fully understanding the underlying concepts and principles (Boursicot et al., 2011; Gormley, 2011). Students also commented that in the OSCE, they would memorize one or two product recommendations that would ensure they could pass; but in real life, there is a wide variety of products to pick from and patient preferences change. While case realism was achieved in the OSCEs, it is still incredibly challenging to replicate a real-life scenario. Students commented that real-life patients don’t always accept your recommendations, you have less time with each patient, there are other factors such as a busy workplace that impact on your time with the patient and you never ask as many history taking questions as you would in an OSCE. The difference in information gathering (Table 3) was markedly different in OSCEs compared to the mystery shopper visit which raises the question whether our OSCE checklists have too many information gathering points which is potentially unrealistic. Others may argue that you need to train students with thorough checklists before they know how to target their questioning.

The extrapolation inference in Kane’s validity framework infers that scores in the assessment should relate to expected competencies in the practical environment and to be aligned with desired learning outcomes (Kane, 1992). Kane’s validity framework emphasizes the importance of multiple sources of evidence, including content, response process, internal structure, and consequences, in establishing the validity of assessment scores, which aligns well with the multifaceted nature of OSCEs commonly used to assess clinical skills and competence (Biggs, 2003). As stated by many in the literature, one of the most challenging obstacles academics face when designing an OSCE is striking a balance between imperfect simulation and fair assessment (ensuring all students in the cohort have the same experience in an exam environment) (Brydges et al., 2015; Gormley et al., 2012; Weersink et al., 2019). Conducting a WBA could combat this dilemma by taking the student out of the exam environment and putting them in the real-life situation. However, WBAs also come with their own challenges. A recent scoping review found that common barriers to workplace based assessment are lack of trainee and assessor engagement in design, time constraints of the clinical environment, and distilling the complex language of competency-based assessment into terms and parameters that assessors and trainees could easily use (Anderson et al., 2021). There are similarities to our study in that some of the main factors contributing to student performance reported by our participants were relationship with workplace supervisor and perceived role and autonomy in the workplace. Having an approachable supervisor or team increased students’ confidence in their therapeutic decision making, and students felt more in control during the OSCEs as opposed to the workplace, where they would have to check with their supervisor when making decisions. Clinical workplace supervisors play a vital role in WBAs and the characteristics of the supervisor can influence learning; supervisors should create learning opportunities and facilitate learning (Keshavarzi et al., 2022). If WBAs are to be integrated, our study team recommends that workplace supervisors are also involved in the OSCE process whether in case design or examination so they have an understanding of the student’s prior knowledge and capabilities. Workplace roles affect student performance, and students noted that patients may place more trust in the pharmacist, which can lead to feelings of incompetence when responding to patient queries or questions during WBAs.

Participants reported feedback from real-life situations (WBAs) was perceived to be more beneficial than feedback from an assessment, but students acknowledged that providing feedback from real-life situations every time would be difficult for assessors. Traditional WBAs such as Direct Observation of Procedural Skills, Mini-Clinical Evaluation Exercise and Case based Discussions involve the workplace supervisor directly observing the encounter with a real-life patient or discussing a case (Liu, 2012). The mystery shopping method, conducted by simulated patients, for WBA would carry less risk than assessing a student using a real patient, and mitigates the risks of consequences of incorrect advice, and repercussions on the workplace/business. Mystery shopping methods have shown its effectiveness in education research (De Almeida Neto et al., 2001) as it can get a true representative of actual behavior at the worksite as well as eliminate Hawthorne effect (Puspitasari et al., 2009). There has been a shift of emphasis from merely assessing behavior of the health professional but also using the outcomes of these visits as formative feedback to enhance continuous professional development (Weiss et al., 2010). This was also echoed by our participants who perceived the feedback from the WBA (mystery shopping visit) more beneficial than the OSCE.

Indeed, as students perceived the mystery shopper to be a real life patient perhaps their motivations changed impacting their performance. As seen in our results, in the OSCE students were more thorough and driven by the goal of grades, whereas in the mystery shopper encounter, the student goal was more to manage the patient efficiently. The activity theory (Vygotsky, 1978) suggests that when the object (e.g., a simulated patient) changes, the goal or motivation for the goal can change, and that the overall activity is “driven by an object-related motive”. Engeström et al. (Engeström, 1999) suggests that activity theory alongside theory of expansive learning provides a conceptual framework for analyzing the complexities and contradictions of systems in health care. Our results from the interviews showed students were concerned about the workplace business, respect for their supervisor and making sure the patient was not inconvenienced and their goals in WBAs were different from their motivation in the OSCE. The healthcare setting is constantly changing and educators need to create innovative solutions to prepare students for the obstacles they may face in real life practice.

### Strengths and limitations

This study has a novel approach of using mystery shoppers in a student assessment context; delivering the same OSCE case in a real life setting which has yet to be conducted in the literature. Mystery shopping has been used to investigate prescribing and pharmaceutical sales provisions (Björnsdottir et al., 2020; Cheo et al., 2020; Collins et al., 2020); but not student competencies. Generally, real life patients are used in WBAs (Khalife et al., 2022; Malau-Aduli, 2022) but we needed to compare performance with the same cases so we trained simulated patients to be used. Due to time constraints and mystery shopper and workplace supervisor availability, it was not possible to visit all the students within their placement period but 92/289 students is a reasonable representation of the cohort. The representation in this sample was more female dominated which is typical of the pharmacy profession (Janzen et al., 2013) and also compounded by the fact more females opted into the study. Depending on time of visit (if visited early on during the placement before a relationship could be established) and work site, students on placement may have had the knowledge to perform but were not sure if they had autonomy to respond to the mystery shopper’s query. Hence, the study complemented the quantitative data with qualitative data to put the scores in context of the students’ bespoke experiences. Another limitation is that this study observes infectious diseases cases only and we hope to extend this to further topics to other clinical scenarios. We could also only practically test their primary care skills with mystery shopping and therefore relied on data from a single OSCE station; future research could look at assessing other pertinent skills such as counseling and medication review to assess students’ overall OSCE assessment performance.

### Educational implications

Academics need to weigh up the logistical challenges of organizing OSCEs and WBAs and consider if an optimal approach of conducting both assessments is practical. From this study, the students valued both assessments and implied they complement each other. Students preparing for an OSCE does result in students memorizing processes and clinical information to regurgitate during the assessment but if complemented with a delayed work based assessment, it forces students to do another re-check that they have retained all the information they learnt and prepared for the OSCE. In this cohort, there was a reliance on references and lack of confidence in their knowledge. Anecdotal evidence from our institution is that relying solely on open book examinations decreases the ability to recall simple concepts confidently; perhaps memorizing information in preparation for an OSCE could improve students’ ability to retain basic clinical concepts. Where possible, academics should involve workplace supervisors in the OSCE process (e.g., case design or examination) to reassure them of the students’ capabilities and to allow them opportunities to practice in the workplace. Further research could explore replicating this study in other disciplines, longitudinally or in other settings e.g. hospital, general practice. This study has shown some challenges to consider when testing the extrapolation inference by Kane, showing factors such as unfamiliarity of the workplace, different patient dynamics and presence of external environment distractions impacting on a students’ performance to perform at their peak level in real life clinical practice. In addition, the weeks leading up to an OSCE is generally filled with immense preparation on the student’s behalf so their scores will often reflect an inflated score showcasing maximum performance competency level, as opposed to the reality of real-life practice.

### Electronic supplementary material

Below is the link to the electronic supplementary material.

Supplementary Material 1

Supplementary Material 2
