# Supplementary material for: Linking assessment to real life practice – comparing work based assessments and objective structured clinical examinations using mystery shopping
Source: Adv Health Sci Educ Theory Pract. 2023 Sep 20;29(3):859–78. doi: 10.1007/s10459-023-10284-1 (PMC11208193; doi:10.1007/s10459-023-10284-1)
Supplement: Supplementary file 1 — Supplementary Material 1 [file 10459_2023_10284_MOESM1_ESM.docx]

**Appendix 1: Interview guide**

1. Firstly, out of interest, could you tell when the mystery shopper came to visit you and what details do you remember about the visit?
2. How did you find completing the case in real life compared to completing the same case in the OSCE setting?

Prompts:

- What about the real life setting was different or similar?
- How did you feel during the OSCE knowing you were being assessed versus managing a real life patient who needs advice?
- Discuss your OSCE preparation

1. Could you think of any factors that might have influenced your performance in the WBA compared to the OSCE?

Prompts:

- Tell me about your supervising pharmacist
- Tell me about your work colleagues e.g. shop assistants
- Tell me about how the retail space is laid out
- Tell me about the environment with other customers, noise, other distractions e.g. phone

1. Before we get into the feedback session for your particular case, we would like to check if knowledge retention was a main factor that affected your performance. I’m going to summarize the case with you now and ask you what pertinent questions you would ask to ascertain the information needed for your recommendation and tell how you would manage the patient.
2. *(Prior to interview, interviewer to listen to audio recording of student’s WBA performance)* Interviewer to summarize the student’s performance in WBA (based on the audio recording) and to pinpoint sections in the interaction that were done well or not so well and ask them to elaborate on why they thought they did well or not.

Prompts exploring poor performance: (If relevant to their performance)

- Comment on poor history taking – being too quick, being too conscious of the time in retail setting, or not engaging in history taking at all
- Comment on the reliance of others - needing to seek help from their preceptor (did they really need help or was it a sign of respect or were they told to ask)
- Comment on communication of sensitive topics to a real life patient - e.g. avoidance of certain history taking questions like sexual health questions
- Comment on the poor closing of the interaction - e.g. No monitoring or follow up advice
- Comment on lack of confidence in recommending a product
- Comment on active listening/not listening to the patient/empathy
- Comment on rushed communication – trying to end the whole conversation very quickly/ brief advice, lack of non-pharmacological advice
