# Supplementary material for: Linking assessment to real life practice – comparing work based assessments and objective structured clinical examinations using mystery shopping
Source: Adv Health Sci Educ Theory Pract. 2023 Sep 20;29(3):859–78. doi: 10.1007/s10459-023-10284-1 (PMC11208193; doi:10.1007/s10459-023-10284-1)
Supplement: Supplementary file 2 — Supplementary Material 2 [file 10459_2023_10284_MOESM2_ESM.docx]

**Appendix 2: Themes, subthemes and representative quotes identified from interviews with students**

| **Main Theme** | **Subtheme** | **Exemplar Quote** |
| --- | --- | --- |
| The fundamental design of the OSCEs differ from real life | Assessment preparation is different from preparing for practice | “the OSCE situation. I guess there is a lot because you're going in with that stressed mindset, whereas in the more relaxed sort of real-life scenario like you just treat them like any other customer or any other patient that comes in so it's a bit more relaxed setting where I feel like I can talk more freely, more easily less structured, and more conversation” [19]  “It kind of helped my confidence to so like knowing that, you know, when I was doing my OSCE it felt like more of an exam situation. So, it felt like I needed to be in a different mindset versus doing it in like the real world, you know” [23]  “I found in the OSCE I'm more prompted to ask, like the referral questions. But in real life I forgot about asking the referral questions, because when I asked about the symptoms they already sounded like referral symptoms, so I didn't like bother checking if they had other referral symptoms” [35] |
|  | Case realism was achieved and the assessment cases do reflect real life cases | “Kind of similar to the OSCE. They kind of marry it all together”[01]  “It's just like the OSCEs…It's just like normal cases, we probably get every day at work” [21]  “I feel like it's [WBA] was the same case as my OSCE…which I can remember that off the top of my head” [30] |
|  | The exam environment is not realistic | “I feel like the exam situation is more stressful”[06]  “Like for all OSCEs I can prepare like there is some time to prepare like my answers for that. But in real like this situation I can really handle that” [09]  “I think the only thing that made me more confident was the fact that I was sort of just not under exam pressure… I could sort of take control of how I wanted to run that interaction….I could do it the way that I wanted to do it rather than you know what sort of expected of you in a in an exam situation. So I think that was the only thing that made it easier, and made my performance better…”[13] |
|  | Feedback from a real-life scenario is more valuable | “a real setting and more practice this way and getting feedback through real life. Practice is so much more helpful… because it’s a very targeted approach” [04]  “I think it's super helpful, because it gives you the chance to kind of reflect as well on what you've done, and how far along you've come through as well. So actually think it was really good, and I found it beneficial” [14]  “I think it's helpful. I guess I can see what my performance is during placement…I asked the pharmacist for feedback. But like the patient's perspective… that’s kind of important. Yeah” [15] |
|  | The simulated patient is not perceived to be real | “In a real life situation, I think it was just a lot more natural… I'm not going to get assessed on this, but I need to make sure that this patient is still getting the correct information, because that is a lot more important than getting a grade…you want to be providing the right information to patients… so I suppose in that aspect. It [WBA] was a little bit more serious” [10]  “Case was similar to OSCE and they spoke more than most usual customers” [12]  “I thought that was very specific, so probably a little bit more detail than the average patient. Or maybe they were wanting to ask a few more questions or listen a bit longer” [14] |
| The work environment has intrinsic factors impacting on the student behavior and performance, meaning simulation can never be fully replicated | The work supervisor or team can impact on WBA performance | “it's helpful to have you know the supervisor or the pharmacist there to sort of refer to if needed” [02]  “if I didn't know how to answer right off the back ,the patient asked me the supervisor or the pharmacist will be the best one of the fastest resources I can have access to” [07]  “I still feel like a little bit unsure of things, just double checking. I feel like I know already, but I just sometimes need to double check with the pharmacists, and it really shows me about my decision, or else I’d be much more hesitant…”[17] |
|  | Their perceived role in the workplace can impact on WBA performance | “ I think the only thing that holds me back is, you know i'm only here temporarily” [01]  “There was a customer that was aggressive and out because they know I'm still a student…they didn't really respect the students. Then they insisted on speaking to the pharmacists, because they know it's just a student, and they don't talk to me…”[33] |
| Students have individual learning characteristics that affect the change in performance in WBA and OSCEs | Students who are concerned about their communication skills are even more impacted in real life | “I don't know if my counseling is good or not so it makes me nervous to approach patients” [33]  “I'm still not really that confident in counseling in English and am worried patients won’t understand me and will do the wrong thing" [36] |
|  | Students who have had previous or current work experience in pharmacy feel more confident with dealing with real life cases | “Having a part time job in a pharmacy definitely made it a little bit easier, you know, having had something similar in OSCE, and then obviously having studied the content more and plus I have a job now in community pharmacy as well and have seen similar cases” [14]  “I think when I'm working like in my regular community pharmacy job. I definitely have more opportunities with to speak to patients. But on placement I didn’t have as many opportunities. Yeah, I guess it's hard because you sort of there for a short period of time, and depending on the time of year. If they're super busy, you know it's a little bit harder to [get support]. But I’m glad I had some”[22] |
|  | Students are still unfamiliar with teaching content even after the assessment | “I had to like research my stuff, I was like, oh, I'll double check because I was probably 90% confident, but it was like I should double check, because she's pregnant and I need my recommendations to be safe” [24]  “I still feel a little bit unsure of things…I just like to double check with the pharmacists” [17] |
